# Urbanized White Ibises (*Eudocimus albus*) as Carriers of *Salmonella enterica* of Significance to Public Health and Wildlife

**DOI:** 10.1371/journal.pone.0164402

**Published:** 2016-10-21

**Authors:** Sonia M. Hernandez, Catharine N. Welch, Valerie E. Peters, Erin K. Lipp, Shannon Curry, Michael J. Yabsley, Susan Sanchez, Andrea Presotto, Peter Gerner-Smidt, Kelley B. Hise, Elizabeth Hammond, Whitney M. Kistler, Marguerite Madden, April L. Conway, Tiffany Kwan, John J. Maurer

**Affiliations:** 1 Warnell School of Forestry and Natural Resources, University of Georgia, Athens, Georgia, United States of America; 2 Southeastern Cooperative Wildlife Disease Study, Department of Population Health, College of Veterinary Medicine, University of Georgia, Athens, Georgia, United States of America; 3 Institute for Environment and Sustainability, Department of Zoology, Miami University, Columbia, Ohio, United States of America; 4 Department of Environmental Health Science, University of Georgia, Athens, Georgia, United States of America; 5 Department of Infectious Diseases, College of Veterinary Medicine, University of Georgia, Athens, Georgia, United States of America; 6 Department of Geography, University of Georgia, Athens, Georgia, United States of America; 7 Centers for Disease Control and Prevention, U.S. Department of Health and Human Services, Atlanta, Georgia, United States of America; 8 Lion Country Safari Park, Loxahatchee, Florida, United States of America; 9 Poultry Diagnostic and Research Center, Department of Population Health, College of Veterinary Medicine, University of Georgia, Athens, Georgia, United States of America; Cornell University, UNITED STATES

## Abstract

Worldwide, *Salmonella* spp. is a significant cause of disease for both humans and wildlife, with wild birds adapted to urban environments having different opportunities for pathogen exposure, infection, and transmission compared to their natural conspecifics. Food provisioning by people may influence these factors, especially when high-density mixed species flocks aggregate. White Ibises (*Eudocimus albus*), an iconic Everglades species in decline in Florida, are becoming increasingly common in urbanized areas of south Florida where most are hand-fed. We examined the prevalence of *Salmonella* shedding by ibises to determine the role of landscape characteristics where ibis forage and their behavior, on shedding rates. We also compared *Salmonella* isolated from ibises to human isolates to better understand non-foodborne human salmonellosis. From 2010–2013, 13% (n = 261) adult/subadult ibises and 35% (n = 72) nestlings sampled were shedding *Salmonella*. The prevalence of *Salmonella* shedding by ibises significantly decreased as the percent of Palustrine emergent wetlands and herbaceous grasslands increased, and increased as the proportion of open-developed land types (e.g. parks, lawns, golf courses) increased, suggesting that natural ecosystem land cover types supported birds with a lower prevalence of infection. A high diversity of *Salmonella* serotypes (n = 24) and strain types (43 PFGE types) were shed by ibises, of which 33% of the serotypes ranked in the top 20 of high significance for people in the years of the study. Importantly, 44% of the *Salmonella* Pulsed-Field Gel Electrophoresis patterns for ibis isolates (n = 43) matched profiles in the CDC PulseNet USA database. Of these, 20% came from Florida in the same three years we sampled ibis. Importantly, there was a negative relationship between the amount of Palustrine emergent wetland and the number of *Salmonella* isolates from ibises that matched human cases in the PulseNet database (p = 0.056). Together, our results indicate that ibises are good indicators of salmonellae strains circulating in their environment and they have both the potential and opportunity to transmit salmonellae to people. Finally, they may act as salmonellae carriers to natural environments where other more highly-susceptible groups (nestlings) may be detrimentally affected.

## Introduction

The genus *Salmonella* has a worldwide distribution and is one of the most common causes of intestinal diseases for both people and animals [1]. Despite major public education efforts and improvements to food hygiene practices, salmonellosis remains a significant source of enteric disease for people in the United States and worldwide. *Salmonella enterica* accounts for 1.2 million illnesses and 450 deaths in the US each year [2]. While most *Salmonella*-associated illnesses had been associated with the consumption of contaminated beef and poultry products [2, 3], there is an increasing frequency of illnesses associated with produce [4–14]. Despite public health education about food handling practices, the incidence of salmonellosis in people has remained the same in the last 20 years, and there has been a shift in serotype distribution from foodborne-associated strains towards environmentally-acquired strains, the source of which is not always known [3, 15, 16].

Although salmonellae are important human pathogens, they can also cause small to large-scale mortalities in wildlife species. For example, throughout the world, *Salmonella* outbreaks caused by *S*. Typhimurium have been reported in passerine birds associated with bird feeders, and to a lesser extent, colony-nesting birds (e.g., egrets and herons) [17–25]. There is still much to learn about the epidemiology and impact of salmonellosis on wild birds as the prevalence of avian salmonellosis appear to be on the rise, in some cases causing catastrophic outbreaks, some of which have been implicated with population-level effects [25, 26, 27]. The increase in outbreaks over the last 25 years has caused some to consider it as an emergent disease that may be directly related to anthropogenic activities such as backyard feeding and the use of contaminated habitats [23, 24, 28, 29].

In general, the level and duration of infection and *Salmonella* shedding in wild birds and potential to develop clinical disease is probably similar to domestic poultry but there are some important epidemiological differences. *Salmonella* prevalence of chicks of some colonial nesting species such as herons and egrets is higher compared to adult birds [25]. The immature intestinal flora related to both age and diet, may explain this difference where the infection rate for adults is relatively low (1–2%), but this may also be due to differences in habitat use. For example, adult herring gulls can be transient carriers of *Salmonella* and may serve in its transport while foraging between contaminated and pristine environments [30]. Several studies illustrate a direct correlation between the prevalence of *Salmonella* shedding in wild birds and their proximity to anthropogenic habitats [21, 31–35]. Urbanized birds likely have unique opportunities for enteric pathogen exposure, infection, and transmission due to various factors such as the quality of food and water they consume, the species with which they have direct contact, their consistent aggregations in high numbers in small areas and comparatively sedentary lifestyles [36]. Urbanized species with a potential to be carriers of *Salmonella* should be of special interest because of their frequent contact with the public and their potential to contaminate environments used by people.

Human *Salmonella* cases have been linked to direct or indirect contact with wild birds in several independent reports [17, 18, 29, 35, 37–42]. The majority of those reports relied on post-outbreak surveys and few utilize molecular typing to investigate similarities between human and avian isolates [37]. Therefore, studies that elucidate the phylogenetic, spatial and temporal relationships between human and bird infections and contribute to understanding of the underlying ecological interactions ecological interactions associated with non-foodborne cases of salmonellosis in humans.

The American white ibis (*Eudocimus albus*) is a gregarious member of the Pelecaniformes that forms large nesting colonies in natural wetlands. Ibises feed by constantly probing the substrate with their beaks for aquatic invertebrates, frogs and fish in wetlands, and roost and nest in mixed-species flocks over water bodies [43]. Their reproductive success and population size is highly dependent on food availability and the hydrology of the Florida Everglades Ecosystem, where most ibis breed [44, 45]. In South Florida, the utilization of urban habitats by white ibis has been steadily increasing since the late 1990s [46]. It is likely that a suite of factors, including natural wetland loss, and a constant source of food and water in urban parks promoted through rapid urban expansion in the region is responsible for this new distribution [47, 48]. Ibises are now abundant in neighborhood parks, golf courses and other artificial wetlands, where they have become sedentary, habituated to consuming food provided by people and where they regularly interact directly and indirectly with humans and other urbanized avian species (e.g, various gull species, semi-domesticated and wild ducks such as Muscovy ducks (*Cairina moschata*), and mallards (*Anas platyrhynchos*) (Hernandez, pers observation). In Australia, a related species of ibis (*Threskiornis molucca*) has become similarly habituated to anthropogenic sources of food and has been shown to be a carrier of *Salmonella enterica* [32]. Thus, we consider the American white ibis a good model species for understanding the role of urbanization on *Salmonella* transmission between wildlife and humans. Specifically, we aimed to determine whether the prevalence of *Salmonella* infection of urbanized white ibises in South Florida was related to either ibis behavior or landscape characteristics, and the relationship between *Salmonella* isolated from urbanized white ibises and human salmonellosis cases. Finally, although urbanized ibises primarily forage in urbanized environments during their non-reproductive period (Hernandez, unpublished data), they disperse long distances to natural areas to breed; thus, we sought to understand the role ibises might play in the dissemination of urban-associated salmonellae to those areas.

## Materials and Methods

### Study Sites

**Fig 1:**
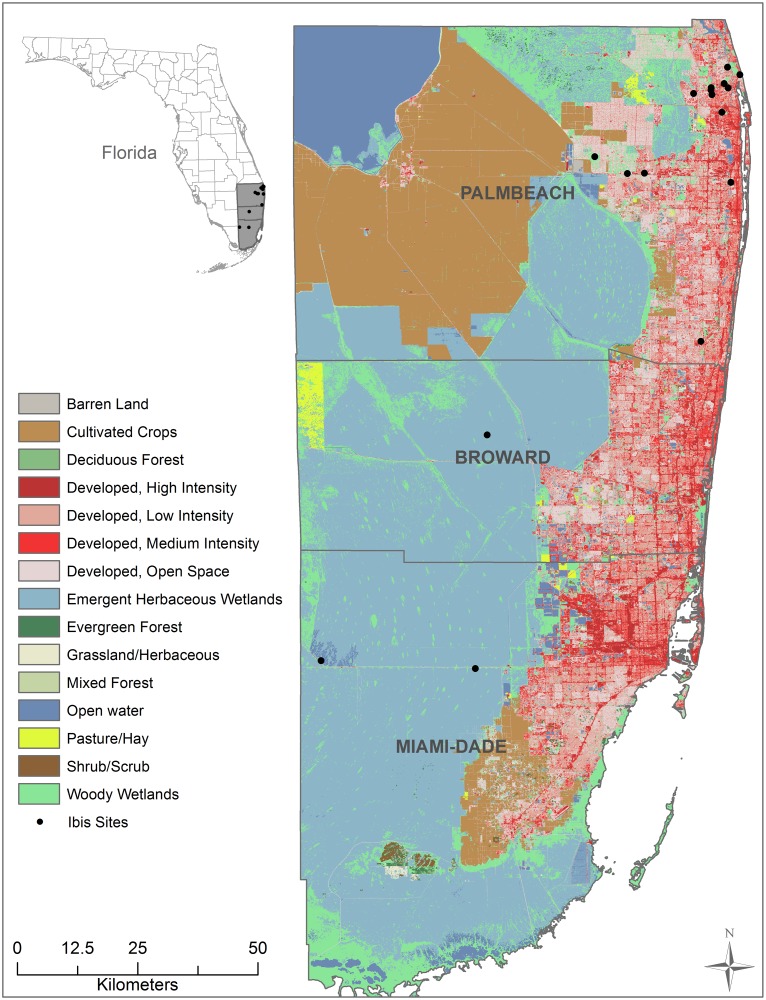
Map of Sites. Fecal samples were collected from adult/subadult and nestling white ibises in Palm Beach, Broward and Miami-Dade counties, FL. The land cover for a 1 km radius surrounding each sampling site was determined using land cover maps.

We collected fecal samples from both adult and subadult birds at 17 public access sites in Palm Beach County, Florida, USA, (26°43’N, 80°2’W) with fresh water bodies from 2010–2013. We defined subadults (and use the term juveniles synonymously) as birds that had left the nest and were less than three years of age, which were readily distinguished from adults by feather coloration. Sites were chosen based on accessibility and a repeated record of more than 10 ibises per site during daily visits. Two sites were within natural areas, whereas the remaining 15 were urban parks, zoos or neighborhoods. We also collected feces from nestlings at three ibis rookeries located within Broward (n = 1) and Miami-Dade (n = 2) counties (Fig 1).

Using ArcGIS version 10.1, the land cover types were determined in a 1 km radius buffer around each site. Within each 1 km buffer, we quantified the percentages of each land cover class to examine the development of land and to classify sites by level of urbanization in each area. The publically-available land cover classes were developed by the Department of Commerce (DOC), National Oceanic and Atmospheric Administration (NOAA), National Ocean Service (NOS), Coastal Services Center (CSC) (http://gis.ncdc.noaa.gov/geoportal/catalog/search/resource/details.page?id=gov.noaa.ncdc:C00814). The NOAA classification originally contained 25 classes, and the accuracy of classification for this data set is 86.5% and 85.1%. Initially, the studied areas represented 18 land cover types ranging from urban areas, agriculture, forested areas, and wetlands (Table 1). However, because some land cover types were <1% of the total land cover for that site, we only included 12 land cover classes in the analysis and excluded Cultivated/Crops, Pasture/Grasslands and Bare Land. For clarification, we defined the four developed land cover types as follows: (1) Developed High Intensity- contained little or no vegetation, including heavily built-up urban centers as well as large constructed surfaces in suburban and rural areas-large buildings such as multiple family housing, hangars, and large barns, interstate highways, and runways, impervious surfaces accounted for 80–100 percent of the total cover (site range = 0 to 18%, Table 1); (2) Developed Medium Intensity- contained substantial amounts of constructed surface mixed with substantial amounts of vegetated surface, includes small buildings such as single family housing, farm outbuildings, and large sheds, impervious surfaces account for 50–79 percent of the total cover (site range = 0.1 to 49%, Table 1); (3) Developed Low Intensity- contained constructed surface mixed with vegetated surface, this class includes features seen in class 2, with the addition of streets and roads with associated trees and grasses, impervious surfaces account for 21–49 percent of the total cover (site range = 0.4 to 40%); and (4) Open Developed- included areas with a mixture of some constructed materials, but mostly vegetation in the form of lawn grasses, this subclass included parks, lawns, athletic fields, golf courses, and natural grasses occurring around airports and industrial sites, impervious surfaces accounted for less than 20 percent of total cover (site range = 0 to 20%).

**Table 1:** Percent land cover of selected land use categories within 1km radius circles surrounding sampling site.

	Burns	Donald Ross	Garden	Juno	San Marco	Lion Safari	Loxa-hatchee	PGA	Prosperity	Royal Palm	San Matera	Zoo	Seasons	Horse shoe	ICP	Kissimmee	Dreher
Developed, High intensity	15.79	2.67	7.65	5.90	17.94	0.46	0.43	3.47	16.34	2.75	14.76	8.48	18.23	4.24	4.41	0.00	7.05
Developed, medium intensity	44.20	31.44	36.20	26.05	29.24	2.87	13.56	29.18	44.51	30.84	43.31	46.00	36.00	25.34	49.16	0.14	46.29
Developed, low intensity	22.27	26.89	19.18	10.15	19.69	11.69	32.16	25.97	20.29	22.61	24.71	21.41	31.70	40.64	19.81	0.40	22.24
Developed, open	9.29	5.82	4.56	10.89	4.33	6.31	5.68	20.55	3.01	7.62	4.01	5.93	3.87	6.56	9.09	0.00	6.08
Herbaceous grassland	0.00	0.37	0.03	0.11	2.35	4.50	2.87	0.37	0.03	2.75	0.00	0.03	0.00	0.06	1.06	19.32	0.03
Evergreen forest	0.00	0.00	0.00	0.00	0.03	0.69	0.49	0.03	0.09	0.03	0.00	0.03	0.00	0.03	0.03	0.11	0.03
Scrub	0.11	0.29	0.14	0.54	0.17	0.63	0.89	0.43	9.86	0.69	0.11	0.09	0.06	0.03	0.09	7.11	0.09
Forest, palustrine	5.93	20.81	22.84	17.03	8.37	63.57	34.48	12.35	0.92	15.85	9.26	10.52	8.46	20.84	4.82	20.04	10.32
Scrub, palustrine	0.66	2.35	1.49	2.01	1.32	5.68	3.15	0.57	1.00	2.04	0.63	1.18	0.43	0.72	1.75	32.04	1.26
Emergent wetland, palustrine	0.89	3.75	3.41	1.03	5.22	2.64	2.69	2.35	0.63	3.84	0.77	1.32	1.03	0.89	4.16	20.15	1.32
Bare land	0.14	0.09	3.41	0.66	5.07	0.23	2.49	0.00	0.00	8.05	0.00	0.00	0.00	0.00	0.03	0.00	0.00
Open water	0.26	4.67	0.72	24.39	5.50	0.03	0.00	3.90	3.24	2.04	2.29	4.61	0.20	0.69	4.64	0.03	4.70

### Ibis behavior

Ibis behavior may be directly related to pathogen transmission among themselves and between ibis and people. For example, ibises that beg for food get very close to people and defecate in very close proximity to people or on structures used by people. Therefore, ibis flocks of adults/subadults were scored by their behavior based on direct observations on a habituation scale of 1–4, where 1 were the least habituated to humans and 4 were the most (Table 2). We based our scoring system on observations of ibis with the general public. Highly habituated flocks responded to people by moving towards them immediately and even tolerated hand feeding. This score was assigned by observing the flock for 30 min prior to any sampling or manipulation. Highly habituated flocks tended to be larger, were more consistently present at a particular urban site and had more opportunities for direct contact with people. In contrast, flocks considered not to be habituated at all did not tolerate the presence of people and flew away when people got within 10 m of the flock. regardless of whether people offered food or not, varied in numbers of individuals and were not found predictably at a particular site. Flock density could be related to behavior and was calculated by counting the number of individuals in the approximate area actively utilized by the flock.

**Table 2:** Habituation behavioral scores assigned to white ibises foraging in urban parks in south Florida.

Score	Definition
**1**	Birds do not allow humans within 10 m and fly off if humans get within that distance
**2**	Birds allow humans within 10 m. but do not associate humans as a source of food, consume food provided by people or beg for food
**3**	Birds will allow humans within 10 m and will consume food provided by humans but do not allow humans within 10 ft. or beg for food
**4**	Birds allow humans within 3 m. and beg for food

### Fecal sample collection and *Salmonella* isolation

The collection of feces from ibises was achieved in one of two ways. In some cases, individual animals (adults/subadults) were observed until they defecated, at which point a sample from the center of the fecal pellet was immediately collected from the ground. Care was taken not to resample individuals by separating one individual from the rest of the flock with a food reward until it defecated. In some instances, as part of a larger project, all nestlings and some adult or subadult birds were individually captured and physically restrained for other procedures, during which time they defecated on a clean, water repellent cloth placed across the holder’s lap. Adult ibises were captured with leg lassos. Briefly, a leg lasso with a slip knot was constructed out of nylon monofilament and hidden in the substrate (i.e. grass). One person would hold the line approximately 6 m away, while a second person would bait a bird close with food, and encourage it to step into the lasso, at which point the line holder would swiftly tighten the lasso, capturing the bird by the leg. Both people would immediately physically restrain it and place it in a pillow case until it was processed. Juvenile ibis were captured prior to their ability to fly by visiting known nests, picking them, placing them in a pillow case and lowering them to the ground where they were immediately processed. In both cases, birds would defecate during handling and approximately 1 g of feces was collected with a sterile swab and immersed in 10 ml of dulcitol selenite broth (Becton, Dickinson and Company, Franklin Lakes, NJ), maintained at room temperature and shipped within 24 h to The Athens Diagnostic Laboratory at the University of Georgia. *Salmonella* enrichment and identification was performed as previously described [49]. Briefly, we performed a double selenite broth enrichment, followed by subculture on XLD and Brilliant Green agar plates (Remel). We selected 5–10 colonies from either selective plate that appeared to be *Salmonella* spp based on morphology. The number of colonies selected was determined based on the numbers of *Salmonella* suspect colonies present. Each colony was then serogrouped. One colony representative from each serogroup was selected and confirmed as *Salmonella* through the following biochemical tests: triple sugar iron (TSI), MIO, citrate, malonate, and phenylalanine. When isolates were confirmed, they were forwarded to the National Veterinary Service Laboratory (NVSL) at Ames, Iowa, for definitive serotyping. No animals were sacrificed as part of this research. All animal handling procedures were approved by the University of Georgia’s Institutional Animal Care and Use Committee (IACUC; A2011 08–018). This work required permits from both the Florida Fish and Wildlife Conservation Commission (LSSC-11-00119F) and the U. S. Fish and Wildlife Service (MB779238-0). Both permits required prior approval by an IACUC. We obtained a Special Use Permit from Arthur R. Marshall Loxahatchee National Wildlife Refuge (B15_09). In addition, for urban parks we obtained permission from the Palm Beach County Parks and Recreations Department. For all sampling on private lands, we obtained permission from the relevant authority. All permits were obtained for the specific procedures of this specific study.

#### Molecular typing of Salmonella isolates by pulsed-field gel electrophoresis

Ibis isolates were strain typed by Pulsed-Field Gel Electrophoresis (PFGE) to determine their genetic relatedness [49] of these isolates to themselves, to archived bird isolates (both wild and domestic), to isolates from other animal sources, to water isolates, and to human isolates deposited in the CDC PulseNet national database which contains >50,000 entries [50]. A master database of *Salmonella* PFGE patterns was generated in BioNumerics (Applied Maths; Austin, TX). This database consisted of 1,047 total PFGE entries for *Salmonella* isolated from water (n = 400) and various animal species (n = 674) and consisted primarily of *S*. *enterica subsp*. *enterica* (97%) isolates representing 58 serotypes. Comparisons were made between PFGE patterns using Dice coefficient and unweighted-pair group method using average linkage (UPGMA) clustering [51].

### Statistical analysis

All data analyses were performed using R version 3.0.2 [52]. For the samples from adult/subadult ibises, generalized linear mixed models (GLMM) in the ‘lme4’ package of R [53]were used to analyze if *Salmonella* prevalence was influenced by the following fixed effects: (1) ibis flock density, (2) the behavioral variable ‘habituation score’, (3) sampling year, and (4) season. All models included site and sampling period as random variables, and used a binomial distribution. A likelihood ratio test was used to compare the GLMM models with and without the fixed effect, i.e. predictor variable, to test for the variable’s significance. In addition to adults and subadults, we also sampled nestlings for *Salmonella*, but in a more limited fashion, i.e. all nestlings were tested during the same season (April/May), and the majority of nestlings were sampled in 2013. Nestlings also did not flock and we were unable to rate habituation scores for adult birds at the rookeries; therefore, we did not test these fixed effects against all three age classes. We were able, however, to test whether there was a difference in *Salmonella* prevalence rates among the three age classes sampled: adults, subadults and nestlings using a GLMM with a binomial distribution. For those predictor variables showing a statistically significant relationship with *Salmonella* prevalence, we calculated the marginal R^2^, i.e. the R^2^ value for only the fixed effects in the model using the package MuMIn in R [54].

To determine the effect of land use/land cover on *Salmonella* prevalence in adult/subadult birds, GLMMs were also used. All models included site and sampling period as random variables, and used a binomial distribution. Land use/land cover categories tested as fixed effects included all twelve of the aforementioned. In addition, due to the lack of predictive power for many of the land cover variables when analyzed separately, we also analyzed the effect of the landscape composition on *Salmonella* prevalence by combining the 12 land cover types into a principal components analysis (PCA), using the function prcomp in R [55]. PCA collapsed the land cover variables into three PCs that explained 73% of the landscape composition (PC1 = 41%, PC2 = 20%, PC3 = 12%). PCA1 loaded negatively on the percentage of land in the four developed categories. PCA2 loaded negatively on the amount of forested areas (Palustrine forest and Evergreen forest) in a landscape (96%). PCA3 loaded strongly and positively on the amount of open water and the amount of open developed land cover. We used GLMMs with a binomial distribution and likelihood ratio tests to evaluate the relationship between the principal components metrics and two response variables: (1) the number of different serotypes and (2) *Salmonella* prevalence. For landscape variables showing a statistically significant relationship with *Salmonella* prevalence, we also calculated the marginal R^2^. We had an *a priori* expectation that there would be a significant difference in *Salmonella* prevalence between adult/subadult and nestlings; therefore, landscape analyses for prevalence did not include the sites from which nestlings were sampled.

To examine whether *Salmonella* prevalence displayed a spatial pattern among the sampled sites (excluding rookeries), we performed a Moran’s I test using a Euclidean distance matrix and the variable *Salmonella* prevalence, using the package spdep in R [56]. To test for patterns of serotype composition by site, we performed a distance based redundancy ordination using the function capscale in the vegan package of R [57]. We examined the various landscape and bird predictor variables (e.g. habituation score) in the ordination. Only sites with at least one ibis testing positive for *Salmonella* were used in this analysis. As we were unable to rate habituation scores for nestlings at the rookeries, these sites were also excluded from this analysis.

To evaluate the potential effects of land use/land cover on the number of isolates from white ibises that matched human cases in the PulseNet database we again used GLMMS with a binomial distribution and likelihood ratio tests. In this analysis we included isolates from nestlings, as we had no *a priori* reason to expect differences among age classes. Land use/land cover categories tested as fixed effects included all twelve of the aforementioned. Marginal R^2^ values were calculated for all significant land use/land cover predictors.

## Results

### *Salmonella* prevalence in Ibis

From January 2010 to July 2013 we collected and tested 261 fecal samples from birds at urban sites and 72 samples from nesting sites. We isolated *Salmonella* from 55 individual birds (33 adult/subadult birds, and 22 nestlings). The mean prevalence for birds (adult/subadult) by site was 13% (range 0–50%) and the mean prevalence for nestlings was 35% (range 6–50%) (Table 3). *Salmonella* prevalence was highest for nestlings, second highest for juvenile or subadult birds, and lowest in adult ibis (likelihood ratio test = 16.99, *P* < 0.001).

**Table 3:** Sampling sites, numbers of birds sampled and overall prevalence of *Salmonella* infection of white ibises in south Florida.

Site	Dates Sampled	N	*Salmonella* prevalence %
Juno Beach urban park	1/2010; 3/2012; 12/2012	45	16
Horseshoe Acres neighborhood	1/2010	9	0
Dreher urban park	1/2010; 3/2012	12	17
Prosperity Oaks retirement community	3/2010; 3/2012; 12/2012	26	15
Garden Lakes business park	3/2010	15	7
Burns Rd urban park	3/2010	5	40
San Matera neighborhood	3/2010	15	13
San Marco Villas neighborhood	3/2010	19	0
Royal Palm urban park	3/2010	10	0
PGA golf community	3/2010	5	40
Donald Ross Rd empty lot	3/2010	10	20
Seasons 52 restaurant	3/2010	10	0
Indian Creek urban park	12/2012	10	10
Lion Country Safari park	3/2010; 3/2012; 12/2012	29	14
Palm Beach Zoo	7/2010; 3/2012	13	8
Loxahatchee National Wildlife Refuge	3/2010	13	0
Kissimmee Prairie Preserve State Park	3/2013; 7/2013	15	27
Tamiami West Colony	4/2012; 4/2013	60	33
Alley North Colony	4/2014	15	6
Ibis Colony	4/2013	28	50
6^th^ Bridge Colony	4/2012; 5/2014	4	50

Combining all three years of the study, habituation score was not a significant predictor of *Salmonella* prevalence (likelihood ratio test = 0.17, *P* = 0.68). The prevalence in 2010 was significantly lower than in 2012 (likelihood ratio test = 8.01, *P* = 0.005), and although there was a trend towards a higher prevalence in 2013, the difference was not statistically significant. Although the majority of adult and subadult birds were sampled from December to March in both years, a few birds were sampled outside of this time period. We tested for an effect of season on *Salmonella* prevalence for adult/subadult birds and found no effect (likelihood ratio test = 0.20, *P* = 0.90). All nestlings were sampled in the same season, April/May. There was no relationship between prevalence and flock density (i.e. the number of white ibis per m^2^) (likelihood ratio test = 1.89, *P* = 0.17); however, for the density calculation we were not able to consider habitat quality.

### Land cover and *Salmonella* prevalence in Ibis

**Fig 2:**
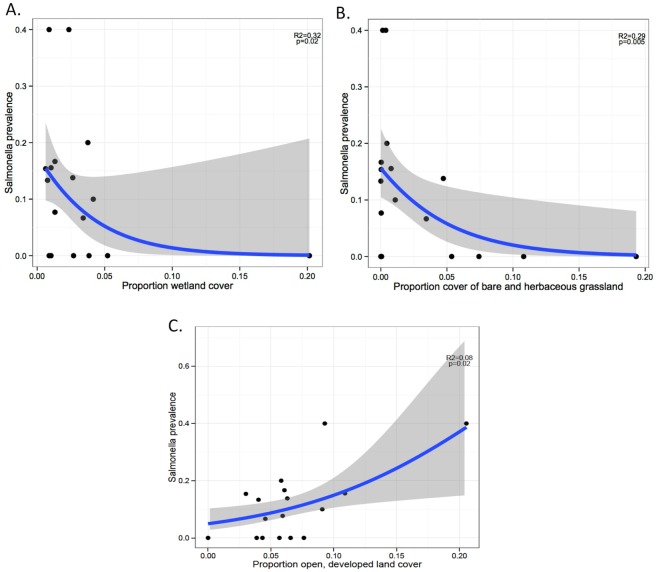
*Salmonella* prevalence and habitat type. *Salmonella* prevalence in white ibises is negatively influenced by cover of emergent wetlands (a) herbaceous grasslands (b) and positively influenced by open, developed land (c) in Palm Beach County, Florida.

Landscape composition metrics determined from PCA were not significantly related to either *Salmonella* prevalence or serotype diversity (numbers of *Salmonella* serotypes isolated; PC1: prevalence, z = 1.04, *P* = 0.29; serotype diversity, z = -0.77, *P* = 0.44). No spatial pattern was found in *Salmonella* prevalence among the sampled sites (Moran’s I: observed = -0.04, expected = -0.06, *P* = 0.90). However, several land cover variables, when examined individually, showed a statistically significant relationship with the prevalence of *Salmonella* infection. As the percent cover of Palustrine wetlands and herbaceous grasslands in a 1km buffer surrounding the sampling site increased, the prevalence of *Salmonella* decreased (wetlands: LRT = 5.41, P = 0.02, grassland: LRT = 8.45, P = 0.004, Fig 2a, 2b and 2c). Palustrine wetlands are non-tidal wetlands dominated by trees, shrubs, and persistent emergent vegetation and low salinity. Within a 1 km radius around each capture point the amount cover of Palustrine emergent wetland ranged from 0.6% to 20% (Table 1). Conversely, the only developed land use type that was related to *Salmonella* prevalence, was the least intensive of the developed cover types, Open Developed, which was positively related to prevalence (Fig 2c, LRT = 5.51, P = 0.02).

### Linkage between *Salmonella* strains in ibises and cases of human salmonellosis

**Fig 3:**
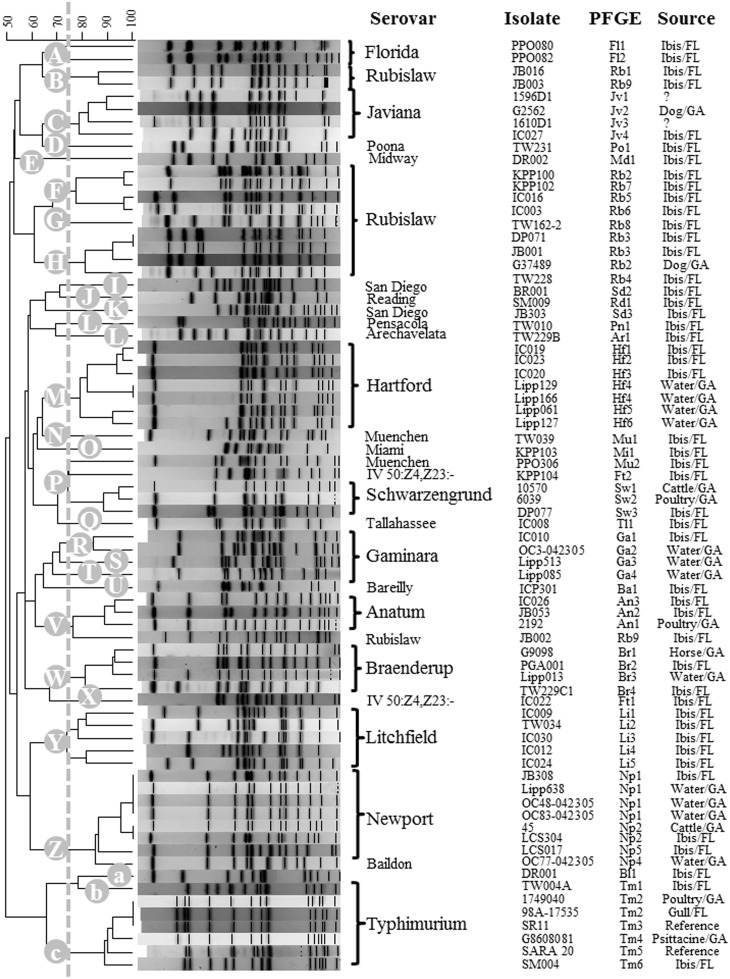
Cluter analysis of Salmonella. Cluster analysis of *Salmonella* isolated from ibises by pulsed-field gel electrophoresis (PFGE) patterns generated with the restriction enzyme *Xba*I. Tiff images of *Salmonella* PFGE patterns were compared using DNA pattern recognition software, BioNumerics (Applied Maths, Austin, TX). Level of similarity was calculated using the band-based Dice similarity coefficient, and clustering of samples was performed using the unweighted pair-group method with arithmetic averaging (UPGMA). *Salmonella* PFGE patterns, generated in this study, were compared to a BioNumerics database of PFGE entries of *Salmonella* isolates from water and various animal species. PFGE patterns generated for ibis isolates were compared against an in-house database of *Salmonella* PFGE profiles for isolates from water or other animal species. A dendrogram was generated of PFGE patterns including those from isolates with the same or similar pattern (≥75% similarity). Clusters with >75% similarities were labeled A-Z, a-c on dendrogram tree on the left.

A high diversity of *Salmonella* serotypes (24 serotypes) and strain types (43 PFGE types) were isolated from white ibises (Table 4). These 43 *Salmonella* strain types belonged to one of 28 clusters identified by PFGE (≥75% similarity; Fig 3). We used rarefaction analysis to determine our sampling effort of serotype diversity in white ibis, which predicted that if another 32 *Salmonella*-infected ibis were tested, between 0 and 22 new serotypes would be found (S1 Fig). Thirty-three percent of the ibis isolates were serotypes that ranked in the top 20 *Salmonella* serotypes associated with human cases in the U.S., as reported by the CDC for years 2010–2012 (*S*. Anatum, *S*. Bareilly, *S*. Braenderup, *S*. Javiana, *S*. Muenchen, *S*. Newport, *S*. Saint Paul, *S*. Typhimurium) [16]. Fifteen percent of our isolates were serotypes most frequently reported in the state of Florida for the years of this study (*S*. Javiana, IV 50:Z4,Z23:- (formerly *S*. Flint), *S*. Newport, *S*. Saintpaul, and *S*. Typhimurium).

**Table 4:** *Salmonella* serotype and strain diversity isolated from American white ibises in South Florida.

Site ^a^	Date	Source	Age ^d^	Isolate	Serotype	PFGE Type	PulseNet	Matches ^h^	Cases in FL ^h^
Kissimmee Prairie Park	07/13	Ibis	Juvenile	KPP100	Rubislaw	Rb2	No Matches		
Kissimmee Prairie Park	07/13	Ibis	Juvenile	KPP102	Rubislaw	Rb7	No Matches		
Kissimmee Prairie Park	07/13	Ibis	Juvenile	KPP103	Miami	Mi1	TEAX01.0045	1	1
Kissimmee Prairie Park	07/13	Ibis	Juvenile	KPP104	Flint	Ft2	No Matches		
Indian Creek Park	12/12	Ibis	Adult	ICP301	Bareilly (10)^e^	Ba1	JAPX01.0064	8	1
Donald Ross Lot	03/10	Ibis	Juvenile	DR001	Baildon	Bl1	TDEX01.0001^g^	107	1
Donald Ross Lot	03/10	Ibis	Juvenile	DR002	Midway	Md1	No Matches	0	0
Juno Beach	01/10	Ibis	Adult	JB003	Rubislaw	Rb9	JLPX01.0002^g^	89	38
Juno Beach	01/10	Ibis	Adult	JB001	Rubislaw	Rb3	No Matches		
Juno Beach	01/10	Ibis	Adult	JB002	Rubislaw	Rb9	JLPX01.0002^g^	89	38
Juno Beach	01/10	Ibis	Adult	JB016	Rubislaw	Rb1	JLPX01.0059^g^	86	24
Juno Beach	03/12	Ibis	Adult	JB053	Anatum (17)^e^	An2	JAGX01.0001	104	3
Juno Beach	12/12	Ibis	Adult	JB303	San Diego	Sd3	No Matches		
Juno Beach	12/12	Ibis	Adult	JB308	Newport (3)^e^	Np1	JJPX01.0025	184	0
San Matera	03/10	Ibis	Adult	SM004	Typhimurium (2)^e^	Tm6	JPXX01.0946	96	0
San Matera	03/10	Ibis	Adult	SM009	Reading	Rd1	JLGX01.0012^g^	7	0
Garden Lake	03/10	Ibis	Adult	GL011	Rubislaw	Rb1	JLPX01.0059^g^	86	24
Prosperity Oaks	03/12	Ibis	Adult	PPO 080	Florida	Fl1	No Matches		
Prosperity Oaks	03/12	Ibis	Juvenile	PPO 082	Florida	Fl2	No Matches		
Prosperity Oaks	03/12	Ibis	Unknown	PPO 085	Bareilly (10^e^	Ba1	JAPX01.0064	8	1
Prosperity Oaks	12/12	Ibis	Adult	PPO 306	Muenchen (8)^e^	Mu2	No Matches		
PGA National	03/10	Ibis	Adult	PGA001	Braenderup (11)^e^	Br2	JBPX01.0008^g^	3	1
PGA National	03/10	Ibis	Adult	PGA004	Saint Paul (8)^e^	NT	ND		
Burns Road Park	03/10	Ibis	Adult	BR001	San Diego	Sd2	No Matches		
Burns Road Park	03/10	Ibis	Adult	BR007	San Diego	Sd2	No Matches		
Lion Country Safari	03/10	Ibis	Adult	LCS017	Newport (3)^e^	Np5	JJPX01.0714^g^	0	0
Lion Country Safari	12/12	Ibis	Adult	LCS040	Anatum (17)^e^	An2	JAGX01.0001	104	3
Lion Country Safari	12/12	Ibis	Adult	LCS300	Bareilly (10)^e^	Ba1	JAPX01.0064	8	1
Lion Country Safari	12/12	Ibis	Adult	LCS304	Newport (3)^e^	Np2	JJPX01.0880	2	0
Palm Beach Zoo	03/12	Ibis	Adult	PBZ060.1	Anatum (17)^e^	An2	JAGX01.0001	104	3
Palm Beach Zoo	03/12	Ibis	Adult	PBZ060.2	Baildon	Bl1	TDEX01.0001	107	1
Dreher Park	03/12	Ibis	Adult	DP071	Rubislaw	Rb1	JLPX01.0059^g^	86	24
Dreher Park	03/12	Ibis	Juvenile	DP077	Schwarzengrund	Sw3	JM6X01.0143	1	0
Ibis Colony, Greater Everglades ^b^	04/13	Ibis	Nestling	IC003	Rubislaw	Rb6	No Matches		
Ibis Colony, Greater Everglades ^b^	04/13	Ibis	Nestling	IC008	Tallahassee	Tl1	No Matches		
Ibis Colony, Greater Everglades ^b^	04/13	Ibis	Nestling	IC009	Litchfield	Li1	No Matches		
Ibis Colony, Greater Everglades ^b^	04/13	Ibis	Nestling	IC010	Gaminara	Ga1	No Matches		
Ibis Colony, Greater Everglades ^b^	04/13	Ibis	Nestling	IC012	Litchfield	Li4	No Matches		
Ibis Colony, Greater Everglades ^b^	03/13	Ibis	Nestling	IC016	Rubislaw	Rb5	JLPX01.0273^g^	3	0
Ibis Colony, Greater Everglades ^b^	03/13	Ibis	Nestling	IC019	Hartford	Hf1	JHAX01.0003^g^	0	0
Ibis Colony, Greater Everglades ^b^	03/13	Ibis	Nestling	IC020	Hartford	Hf3	No Matches		
Ibis Colony, Greater Everglades ^b^	03/13	Ibis	Nestling	IC022	Flint	Ft1	TDHX01.0023	1	1
Ibis Colony, Greater Everglades ^b^	05/13	Ibis	Nestling	IC023	Hartford	Hf2	JHAX01.0080^g^	0	0
Ibis Colony, Greater Everglades ^b^	05/13	Ibis	Nestling	IC024	Litchfield	Li5	No Matches		
Ibis Colony, Greater Everglades ^b^	05/13	Ibis	Nestling	IC026	Anatum (17)^f^	An3	JAGX01.0033	15	1
Ibis Colony, Greater Everglades ^b^	05/13	Ibis	Nestling	IC027	Javiana (4)^f^	Jv4	No Matches		
Ibis Colony, Greater Everglades ^b^	05/13	Ibis	Nestling	IC030	Litchfield	Li3	No Matches		
Tamiami W Colony ^c^	03/13	Ibis	Nestling	TW004A	Typhimurium (2)^f^	Tm1	No Matches		
Tamiami W Colony ^c^	03/13	Ibis	Nestling	TW010	Pensacola	Pn1	No Matches		
Tamiami W Colony ^c^	04/12	Ibis	Nestling	TW034	Litchfield	Li2	No Matches		
Tamiami W Colony ^c^	04/12	Ibis	Nestling	TW039	Muenchen(8)^e^	Mu1	JJ6X01.0426^g^	1	0
Tamiami W Colony ^c^	04/13	Ibis	Nestling	TW162-2	Rubislaw	Rb8	No Matches		
Tamiami W Colony ^c^	04/13	Ibis	Nestling	TW229B	Arechavelata	Ar1	AREX01.0029	0	0
Tamiami W Colony ^c^	04/13	Ibis	Nestling	TW229C1	Braenderup(11)^f^	Br4	No Matches		
Tamiami W Colony ^c^	04/13	Ibis	Nestling	TW231	Poona	Po1	JL6X01.0678^g^	1	1

^a^ Sites are ordered in table corresponding to location from north (top) to south (bottom).

^b^ Miami, Dade County.

^c^ Ibis colony located in Broward County.

^d^ Juveniles were identified by plumage color and were defined as birds < 3 years of age. Adults: >3 years of age. Nestlings were defined as birds that remained in the nest up to 25 days of age.

^e^ Ranking of *Salmonella* serotype associated with human cases reporting for year of isolation in the U.S.

^f^ *Salmonella* serotype in numerical ranking, in human cases for last year reported in in 2012.

^g^ PFGE pattern not associated with any foodborne outbreaks for year of isolation.

^h^ Matching entries for PFGE pattern for year of isolation.

*Salmonella* serotypes Anatum, Braenderup, Hartford, Newport, Schwarzengrund, and Typhimurium from ibises had identical or highly similar PFGE patterns to archived isolates from water or other animal species [49, 58–60] (Fig 3). However, the PFGE patterns for *Salmonella* serotypes *S*. Bareilly, *S*. Muenchen, and *S*. Rubislaw isolated from ibises were distinct (<75% similarity) from PFGE profiles generated for the same serotypes isolated from other wildlife and water sources (data not shown).

**Fig 4:**
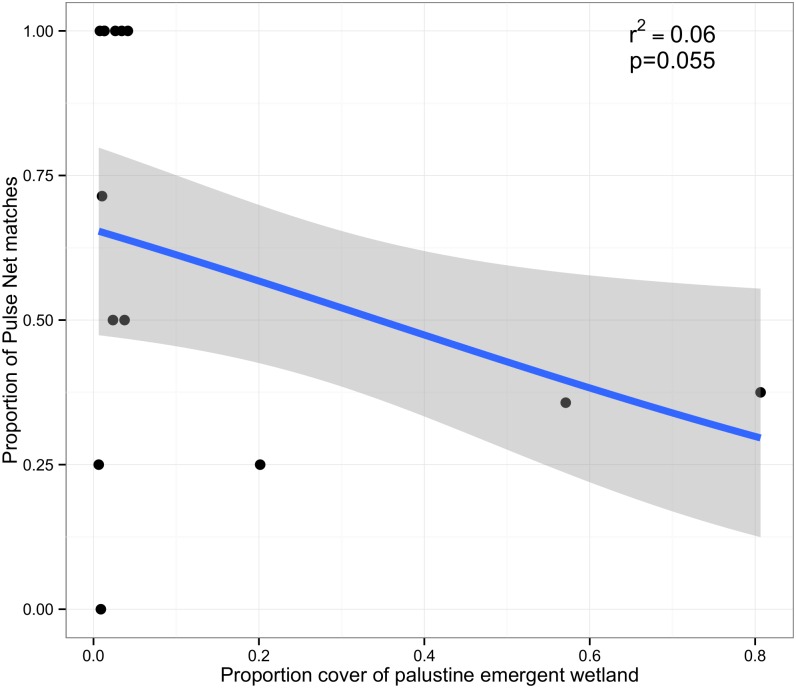
*Salmonella* matches and emergent wetland habitat type. *Salmonella* isolates from white ibises in Florida that matched human isolates in the PulseNet database were negatively, and marginally statistically significantly related with the land cover type Palustrine emergent wetland.

Additionally, forty-four percent of the *Salmonella* PFGE patterns for white ibis isolates (n = 43), matched profiles in the CDC PulseNet USA database. Several of these PulseNet patterns have not been associated with any foodborne outbreaks suggesting an environmental source (Table 4). Eighty percent of ibis isolates belonging to one of the top 20 *Salmonella* serotypes reported to the CDC (n = 15), had matching PFGE pattern with PulseNet. Twenty percent of entries reported to PulseNet, with matching patterns (n = 43), had reported human cases in Florida for the year we isolated the strain from ibis (n = 72, years 2010–2013). Florida had the most cases of any state associated with *S*. Rubislaw PulseNet patterns JLPX01.0002 and JLPX01.0059, and accounted for 86% human cases in Florida (n = 72). All *Salmonella* Rubislaw, isolated from ibises, produced unique PFGE patterns compared to PFGE patterns for *S*. Rubislaw isolated in Georgia [1]. Three of the eight white ibis *S*. Rubislaw PFGE patterns matched PFGE patterns for S. Rubislaw in PulseNet database of human cases. A non-random association was also observed in the distribution of *Salmonella* strain types from white ibises that matched human cases when examined by land cover type. There was a negative, but not statistically significant, relationship between Palustrine emergent wetland and the number of *Salmonella* isolates from white ibises that matched human cases in the PulseNet data base (p = 0.055; Fig 4). Furthermore, there was spatial and temporal overlap observed for *Salmonella* PFGE types isolated from ibises and humans in Florida (Table 5).

**Table 5:** *Salmonella* PFGE patterns from American white ibis isolates that matched CDC Pulsenet database and human cases reported in Florida.

Serotype	PulseNet Pattern	Date of Isolation		Florida County	
		Ibis	Human	Ibis	Human
Anatum	JAGX01.0001	03/2012;12/2012	02/24/2014	Palm Beach	Lee
			05/22/2012	Palm Beach	Broward
			11/20/2012	Palm Beach	Indian River
	JAGX01.0033	05/2013	12/28/2012	Dade	Hillsborough
			06/23/2013	Dade	Putnam
Baildon	TDEX01.0001	03/2012	08/07/2010	Palm Beach	Broward
Bareilly	JAPX01.0064	12/2012	08/20/2012	Palm Beach	Broward
Braenderup	JBPX01.0008	03/2010	02/12/2010	Palm Beach	Unknown
IV 50:Z4,Z23:- (formerly Flint)	TDHX01.0023	03/2013	10/31/2013	Greater Everglades	Volusia
Lomalinda	TDEX01.0001	3/2010	08/07/2010	Palm Beach	Broward
Miami	TEAX01.0045	07/2013	06/08/2013	Okeechobee	Pasco
Rubislaw	JLPX01.0059	01/2010; 03/2010	01/14/2010	Palm Beach	Lee
			02/16/2010		Palm Beach
			03/13/2010		Broward
			04/11/2010		Pinellas
			04/24/2010		Palm Beach
			05/08/2010		Orange
			05/29/2010		Orange
			06/12/2010		Palm Beach
			06/16/2010		Broward
			06/27/2010		Broward
			07/08/2010		Broward
			07/13/2010		Orange
			07/14/2010		Hillsborough
			07/18/2010		Palm Beach
			07/30/2010		Broward
			08/02/2010		Orange
			08/19/2010		Brevard
			08/19/2010		Sarasota
			08/23/2010		Broward
			08/28/2010		Hillsborough
			08/31/2010		Pinellas
			09/17/2010		Brevard
			09/21/2010		Hillsborough
			10/08/2010		Seminole
			10/09/2010		Seminole
			10/09/2010		Lake
			11/11/2010		Hillsborough
			11/15/2010		Hillsborough
			12/31/2010		Hillsborough
		3/2012	02/14/2012	Palm Beach	Lee
			02/23/2012		Palm Beach
			03/30/2012		Broward
			04/24/2012		Unknown
			06/07/2012		Hillsborough
			06/09/2012		Pinellas
			06/30/2012		Hillsborough
			07/01/2012		Pinellas
			08/09/2012		Polk
			08/18/2012		Lee
			08/19/2012		Lake
			08/21/2012		Hillsborough
			09/03/2012		Broward
			09/10/2012		Hillsborough
			09/14/2012		Palm Beach
			10/03/2012		Sarasota
			10/06/2012		Broward
			10/15/2012		Polk
			10/20/2012		Pasco
			11/01/2012		Lee
			11/04/2012		Palm Beach
			11/23/2012		Broward
			12/12/2012		Unknown
			12/28/2012		Palm Beach

## Discussion

Previous reports have documented isolated and limited outbreaks of salmonellosis of colony-nesting birds, particularly during die-offs of nestlings [19, 39, 61–63]; however, this is the first report on the prevalence of *Salmonella* spp. in healthy, American white ibises. We found that the prevalence of *Salmonella* in ibises decreased as the percent of Palustrine wetlands and herbaceous grasslands increased, suggesting that land cover types that are closer to natural ecosystems support birds with a lower prevalence of carriage. White ibises are more highly dispersed in these habitats and wetlands/herbaceous grasslands provide high quality foraging habitat. Conversely, the prevalence of *Salmonella* shedding increased with the Open Developed land cover type. Ibises, as well as other peridomestic species known to carry *Salmonella*, seem to be highly attracted to open, developed areas because they are an optimal mixture of anthropogenic materials with high levels of vegetation that promote both foraging in grasses (particularly recently irrigated lawns and golf courses) and receiving handouts in parks and fields [64]. Urban parks in particular, where white ibises congregate in search of food provisions, promote the prolonged use of these sites by birds at high densities of individuals, likely resulting in cycles of *Salmonella* infection within those populations. Congregating around food sources, especially anthropogenic sources, is a well-known risk for transmission of pathogens, including *Salmonella*, among numerous wildlife species [65]. Persistently contaminated environments are a result of both birds that are carriers intermittently excreting salmonellae into the environment, and the environmental persistence of *Salmonella* spp [23, 66]. We found a significantly higher in prevalence in 2012, when compared to 2010, which may be attributed to hydrological conditions and its effect on the foraging behavioral patterns of white ibis. Years of unusually high or low precipitation create situations in natural wetlands that promote the utilization of anthropogenic food sources by white ibises [67] and the year 2012 was among the driest of our sampling period and one that would be consistent with increased urban foraging. While we did not find a positive association between the habituation score (as an indicator of dependency to urban habitats) and *Salmonella* prevalence, it is important to note that our comparison is based a small sample of animals that were not habituated and that behavior among birds may be site-specific.

The high diversity of *Salmonella* serotypes and strains found in white ibises suggests a lack of host-specificity and indicates that white ibises are likely to acquire *Salmonella* from their environment. Similarly, field and experimental studies of adult herring gulls (*Larus smithonianus*) and other urbanized birds demonstrate rapid elimination of *Salmonella* from the intestines, and a clear relationship between human/domestic animal waste and *Salmonella* colonization. This suggests that like these birds, ibis may be transiently colonized by *Salmonella* and may simply serve as a mechanical transport mechanism for the movement of salmonellae ingested from contaminated environments (for example, see Cizek et al.1994 [68], Reche et al 2003 [34] but for a review, see Tizard, 2004 [24]). Of the most frequently isolated serotypes from ibises, *S*. Litchfield, *S*. Rubislaw and *S*. Miami were specifically correlated with wetland land cover types—serotypes that are not among the most frequently reported in humans in Florida or US-wide (S2 Fig).

Twenty-five of the PFGE patterns detected in ibis matched human clinical cases in the PulseNet database. Of those, 72% (n = 18) matched human cases from the state of Florida. There was a negative but only marginally statistically significant relationship between the number of isolates that matched human cases and emergent wetlands. At first glance, it might appear that age influenced this result, as nestlings made up a large proportion of the isolates from that land cover type and had the highest prevalence of infection; however, the nestlings we sampled were still fed solely by their parents who are foraging in nearby available habitat, confirming that the isolates from ibises in natural environments are dissimilar from human isolates.

The Florida Department of Health reports that the majority of the 5,000–6,000 human salmonellosis cases reported every year are sporadic in nature, or not associated with a food outbreak, and the most significant sources of these infections have not been identified to date (Florida Dept of Health, 2012 [69]). In urban parks in Palm Beach County, people actively feed white ibises at very close range (birds take food from people’s hands) and it is common to see people, especially children and the elderly, leaning or sitting on surfaces contaminated with white ibis feces. Specifically in Florida, the state that houses the largest breeding colonies of white ibises in the US, there are over 5,000 cases of salmonellosis every year, of which nearly half are children less than 5 years of age for which consequences of infection can be severe (Florida Bureau of Environmental Public Health Medicine, Division of Environmental Health web 2011 [70]). Ibises have always been a common sight in southern Florida, but their adaption to urban habitats due to wetland loss and the increasing urbanization in Palm Beach County may increase the interaction between ibises and people [71]. Recently, urbanized ibis flocks have been noted in other urban centers in south and central Florida (Hernandez, personal observations). In Australia, highly urbanized ibises of a different species but with similar ecology are described as a nuisance and considered a threat public health threat, in part due to their carriage with *Salmonella* [32, 47]

These results indicate that ibises are likely acquiring salmonellae from environmental sources, may be good indicators of salmonellae circulating in their environment, and have the potential and opportunity to transmit salmonellae to people. Future testing of environmental samples is recommended. Additionally, our preliminary work of radio tagged ibises suggests that ibises tagged at urban sites move among several urban foraging sites on a weekly basis, and exhibit high site fidelity to urban habitats during the non-breeding season (using urban parks on a daily basis), yet fly long distances to return to natural areas to roost at night, such as the Loxahatchee National Wildlife Refuge or back and forth to nest and acquire food for nestlings between March-July in the Greater Everglades Ecosystem (Hernandez, unpublished data) where they can disseminate salmonellae acquired in urban areas. It is at these remote breeding areas that nestling mortality is likely to occur but least likely to be detected. Further research is needed to elucidate the relationship between habitat type, and the rate and persistence of *Salmonella* infection, and strain types.

## Supporting Information

S1 Fig Prediction of serotype diversity. Rarefaction prediction for serotype diversity of *Salmonella* isolated from white ibises in Palm Beach, Florida.
(TIF)

S2 Fig Ordination for serotype diversity. Ordination analysis for serotype diversity and sampling site of *Salmonella* isolated from white ibises in Palm Beach, Florida. The size of the circles varies in size based on the proportion of wetland land cover within a 1 km radius from the sampling site.
(TIFF)
