# Supplementary figures and images for: Urbanized White Ibises (Eudocimus albus) as Carriers of Salmonella enterica of Significance to Public Health and Wildlife
Source: PLoS One. 2016 Oct 21;11(10):e0164402. doi: 10.1371/journal.pone.0164402 (PMC5074519; doi:10.1371/journal.pone.0164402)

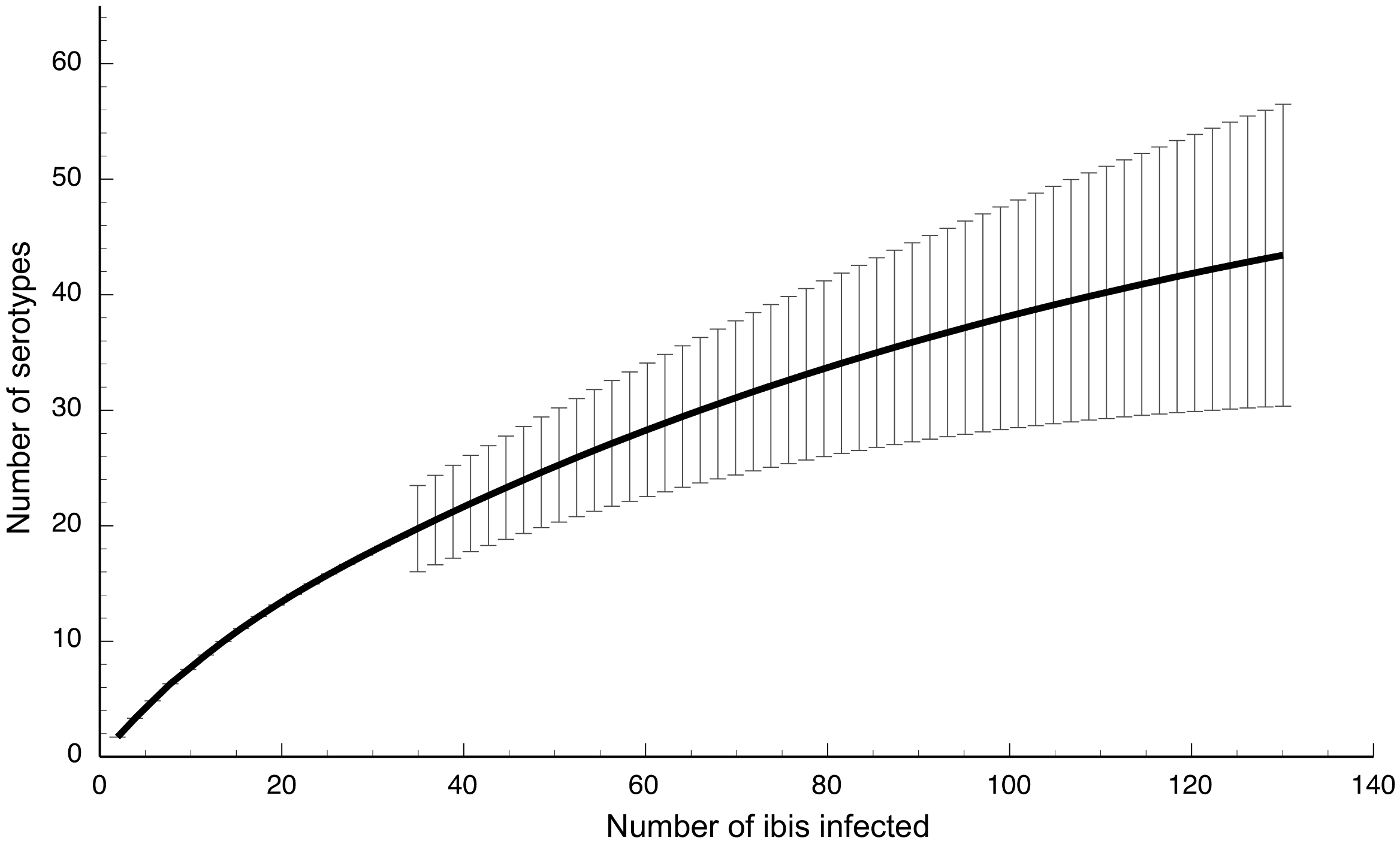

Supplement: S1 Fig — Rarefaction prediction for serotype diversity of Salmonella isolated from white ibises in Palm Beach, Florida. (TIF) [file pone.0164402.s001.tif]

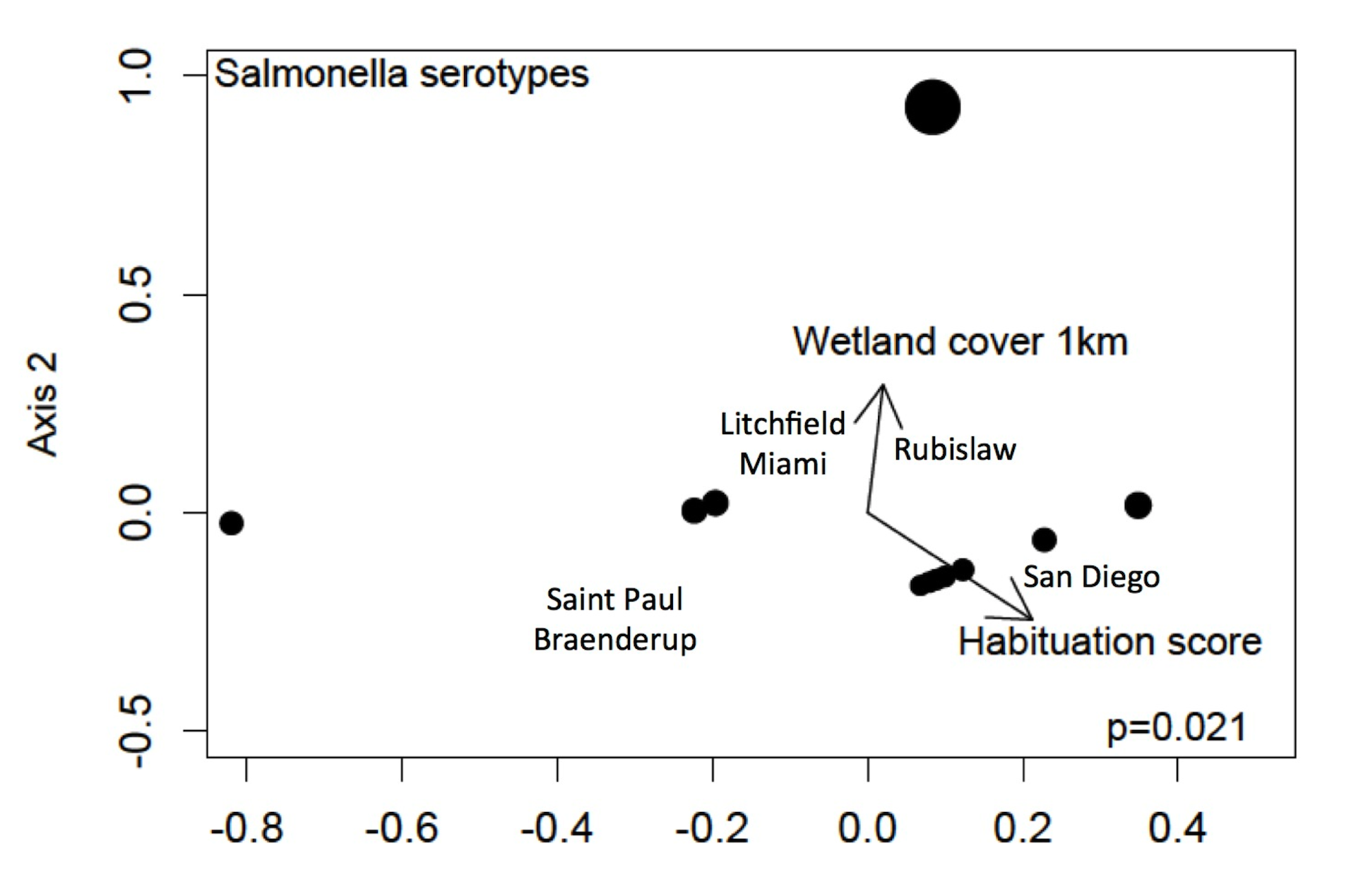

Supplement: S2 Fig — Ordination analysis for serotype diversity and sampling site of Salmonella isolated from white ibises in Palm Beach, Florida. The size of the circles varies in size based on the proportion of wetland land cover within a 1 km radius from the sampling site. (TIFF) [file pone.0164402.s002.tiff]
